# Liver Abnormalities after Elimination of HCV Infection: Persistent Epigenetic and Immunological Perturbations Post-Cure

**DOI:** 10.3390/pathogens10010044

**Published:** 2021-01-07

**Authors:** Stephen J. Polyak, I. Nicholas Crispe, Thomas F. Baumert

**Affiliations:** 1 Department of Laboratory Medicine and Pathology, University of Washington School of Medicine, Seattle, WA 98195, USA; 2 Department of Global Health, University of Washington, Seattle, WA 98195, USA; 3 Department of Microbiology, University of Washington, Seattle, WA 98195, USA; 4 Department of Immunology, University of Washington, Seattle, WA 98195, USA; 5 Institut de Recherche sur les Maladies Virales et Hépatiques, Université de Strasbourg, Inserm U1110, 67000 Strasbourg, France; 6 Pole Hépato-digestif, IHU, Hopitaux Universitaires de Strasbourg, 67000 Strasbourg, France

**Keywords:** hepatitis C virus, DAA, liver cancer, HCC, epigenetic, innate immunity

## Abstract

Chronic hepatitis C (CHC) is a major cause of hepatocellular carcinoma (HCC) worldwide. While directly acting antiviral (DAA) drugs are now able to cure virtually all hepatitis C virus (HCV) infections, even in subjects with advanced liver disease, what happens to the liver and progression of the disease after DAA-induced cure of viremia is only beginning to emerge. Several large-scale clinical studies in different patient populations have shown that patients with advanced liver disease maintain a risk for developing HCC even when the original instigator, the virus, is eliminated by DAAs. Here we review emerging studies derived from multiple, complementary experimental systems involving patient liver tissues, human liver cell cultures, human liver slice cultures, and animal models, showing that HCV infection induces epigenetic, signaling, and gene expression changes in the liver associated with altered hepatic innate immunity and liver cancer risk. Of critical importance is the fact that these virus-induced abnormalities persist after DAA cure of HCV. These nascent findings portend the discovery of pathways involved in post-HCV immunopathogenesis, which may be clinically actionable targets for more comprehensive care of DAA-cured individuals.

## 1. Introduction

Hepatitis C virus (HCV) causes chronic infection and liver disease and infects an estimated 71 million people worldwide [1]. This year, the Nobel Prize for Medicine was awarded to Harvey Alter, Michael Houghton, and Charlie Rice for the discovery of HCV [2]. The award commemorates a decades-long quest; the virus was first described as non-A, non-B hepatitis by Alter and colleagues [3,4], discovered as HCV by Houghton and colleagues [5], and shown by Rice and colleagues that the RNA was infectious in vivo [6], resulting in effective antivirals, and eventually, cures for millions of patients. It was clear from the start that since HCV is an RNA virus that replicates exclusively in the cytoplasm of infected cells, patients could be cured of their infection. Interferon (IFN)-based treatment of HCV [7] was the cornerstone of cures from the late 1970s until about 2010. The landmark studies by this year’s Nobel Laureates, in addition to other major contributions in understanding the enzymatic functions of viral enzymes and virus replication [8] and infection systems [9], led to revolutions in the treatment of HCV infection by directly acting antiviral (DAA) drugs that target the virus-encoded nonstructural (NS) NS3/4a protease, NS5A protein, and the NS5B polymerase [10]. Oral DAA regimens are now available that can cure the majority of HCV infections, regardless of viral genotype and disease severity, two major limitations of earlier IFN-based therapy.

## 2. What Happens to the Liver after Eradication of HCV?

IFN-based cures can reverse liver damage [11]. However, halting or reversing liver disease upon IFN cure of HCV has not been universal; some patients with advanced disease still go on to develop hepatocellular carcinoma (HCC) [12,13,14]. DAA drugs are now able to cure the majority of HCV infections, even in subjects with advanced liver disease [15,16,17]. While it was recently suggested that previously treated HCC can reappear faster or more aggressively in patients who are cured of their HCV infection [18], this highly controversial finding has been refuted by numerous subsequent studies [19,20]. Just like IFN-based treatment and eradication of infection, DAA cure of HCV does not result in increased or accelerated HCC; however, the risk of HCC remains even after the HCV is eliminated [21,22,23]. This review summarizes the emerging information on the molecular underpinnings for the persisting risk of liver disease in DAA-cured patients, with a focus on innate immune, epigenetic, gene expression, and signaling changes induced by HCV and the fate of these virus-induced changes once the virus is eradicated by DAAs.

## 3. Hepatic Immune Abnormalities in Chronic Hepatitis C and the Effects of DAA Cure on Innate Hepatic Immunity

HCV is a highly successful pathogen that subverts both innate and adaptive immune mechanisms in infected hepatocytes, neighboring non-parenchymal cells, and circulating NK cells and T cells. A sure indication of the importance of a host defense mechanism is the precision with which successful pathogens have evolved to evade it. Thus, while human host resistance to HCV, as well as the responsiveness of an individual to DAA therapy, is closely tied to specific alleles of the interferon lamda 3 (IFNL3) gene for subjects with HCV genotype 3 infection [24], the importance of IFNA/IFNB is underlined by the fact that the NS3/4a protease of HCV disrupts two distinct upstream inputs that activate interferon regulatory factor 3 (IRF3) and type 1 IFN. Thus, the recognition of double stranded RNA (dsRNA) by toll-like receptor 3 (TLR3) provides surveillance of intra-vesicular spaces; this TLR engages IRF3 via the Toll/interleukin-1 receptor (TIR) domain-containing adaptor protein inducing interferon beta (TRIF), and this molecule is cleaved by HCV NS3/4a protease [25,26]. Conversely, cytoplasmic space is subject to surveillance by RIG-like receptor (RLR) sensors that signal through mitochondrial antiviral signaling protein (MAVS), and this molecule too is cleaved by HCV NS3/4a [27,28]. It therefore seems likely that, in the right place and at the right time, type 1 IFN is potentially an effective host defense against HCV; however, the virus has effectively blocked this pathway in infected cells. While there is some benefit for a subset of patients in the systemic administration of IFN-alpha, this avenue of therapy is limited by toxicity; adverse effects may be more common in those who achieve a good therapeutic response [29]. Nevertheless, endogenous immunity in peripheral blood mononuclear cells entails a strong type 1 IFN response, which disappears if the virus is cleared [30], along with anti-viral genes and inflammatory chemokines [31].

The genome wide association studies (GWAS) implicating IFNL3 in the response to therapy [32,33], the clinical data on the effectiveness of IFNA/IFNB, and the strenuous efforts made by the virus to disable the signaling pathways leading to IFNA/IFNB responses all argue that the common downstream effects of IFN signaling are important for virus control. While IFNA and IFNB share a family of receptors, signaling via the interleukin 28 receptor (IL28R) involves one unique signaling chain as well as a chain shared with the interleukin 10 receptor (IL-10R) [34]. The expression of the three IFNL genes is induced by signaling pathways overlapping those that activate IFNA and IFNB, namely IRF-3, IRF-7, and nuclear factor kappa B (NF-kB) [35]. Although the cytokines are synergistic in their antiviral effect, no distinct set of interferon-stimulated genes is induced by either cytokine family [36]. However, IFNA induces these genes faster, while expression is more prolonged in the case of IFNL3. This might explain the synergistic effects of the two families of IFNs.

The spontaneous resolution of HCV infection in a minority of patients also sheds light on the mechanisms of host defense. In a study comparing over 900 subjects with evidence of spontaneous HCV clearance and over 1400 chronically infected controls, the same IFNL3 gene polymorphisms were associated with self-cure as with cure by IFNA plus ribavirin [37]. The same polymorphisms influenced the response of HCV genotype 3 to DAA drugs [24].

The innate immune features of chronic HCV that are sustained after self-cure, cure by IFNA plus ribavirin therapy, and cure by DAA therapy have not been extensively documented. A longitudinal study of innate immunity in spontaneous resolution of HCV in intravenous drug users suggested that most innate immune genes in peripheral blood cells returned to baseline by the time of follow-up [30]. However, events in the liver may be different. In liver biopsies, while IFN gamma (IFNG) and IFNL gene expression rapidly disappeared after successful DAA treatment, the level of IFNA2 gene expression was sustained in many patients [38]. More striking was the effect on MAIT cells, where successful DAA treatment failed to restore this population on long-term follow-up [39]. The relationship between these events and HCV-induced, DAA-refractory changes in the phosphoinositide 3-kinase (PI3K) pathway (see below) are currently not understood.

In the face of so many mechanisms through which HCV disables innate immunity, the induction of innate immune genes and cytokines requires explanation. Our own work shows that semi-purified hepatocytes result in a much richer innate immune response to HCV than highly purified hepatocytes [40], implying that it is non-parenchymal cells that drive most of the response. How the signal is passed from the infected hepatocyte to a non-infected and thus non-compromised non-parenchymal cell may involve the transfer of exosomes [41,42,43]. However, such exosomes may also inhibit TLR3 function, impairing effective host defense [44].

HCV also dysregulates innate immune function through effects on cells that are not readily susceptible to infection, i.e., myeloid cells and natural killer (NK) cells. The most abundant myeloid cells in the normal liver, Kupffer cells, are liver-resident macrophages; in mouse models there is strong evidence that many of these cells originate from the yolk sac and the fetal liver [45], while a subset are derived from blood monocytes [46,47]. The abundance of monocyte-derived cells is increased after Kupffer cell depletion, an aspect of emergency myelopoiesis. Two Kupffer cell subsets were resolved in single-cell RNA sequencing analyses of unmanipulated mouse liver [48], and two broadly equivalent subsets were resolved in human liver, both by single cell RNA sequencing [49,50] and by flow cytometry [51]. Current research does not address whether HCV modulates the activity of one or both Kupffer cell subsets.

While the HCV protease exerts immune effects in the infected cell, HCV core protein is well known as a disruptor of immunity [52,53] and has a strong effect on bystander cells. In human Kupffer cells, HCV core binds to TLR2 and activates pro-inflammatory pathways leading to the secretion of IL-1B and TNF-alpha, but also activates immunosuppressive pathways mediated by IL-10 and increased expression of programmed death-ligand 1 (PD-L1). When human Kupffer cells were stimulated via TLR3, they secreted IFNA and IFNB, but the addition of HCV core inhibited these pathways. Critically, the up-regulation of PD-L1 was blocked by an inhibitor of PI3K, but so was the induction of IFNA and IFNB via TLR3 [54]. Thus, this study revealed that both pro-inflammatory and immunosuppressive effects of HCV core were mediated via PI3K. In a follow-up study, human monocytes exposed to either HCV core or infectious HCV virions adopted additional features of myeloid-derived suppressor cells, including the induction of indoleamine-pyrrole 2,3-dioxygenase (IDO-1) and its product kynurenine; this was also dependent on TLR2, protein kinase B (PKB), PI3K, and signal transducer and activator of transcription 3 (STAT3) signaling [55].

Monocytes from HCV+ individuals contain detectable HCV RNA, but this is lost as they differentiate into dendritic cells [56]. The HCV core protein also acts on human monocytes in vitro, impairing their differentiation into dendritic cells and instead promoting the macrophage cells fate. Such HCV-influenced monocyte-derived macrophages polarize CD4+ T cells towards the Th-17 fate, rather than the Th-1 fate that is more effective in anti-viral immunity [57]. Several other HCV-encoded proteins also subverted the functional maturation of, and T cell stimulation by, dendritic cells [58]. When human blood monocytes were polarized in vitro to either the M1 or the M2 differentiation state, HCV core impaired both maturation processes. Strikingly, blood monocytes from chronically HCV-infected patients were enhanced in the expression of both M1-specific and M2-specific genes in the presence of a DAA that inhibited NS5a, showing that the catalytic action of the viral enzyme was required for such immune subversion [59].

HCV also exerts powerful effects on, and appears to be controlled in part by, NK cells [60]. A lower frequency of NK cells in peripheral blood was associated with persistent infection in a group of patients infected from a common source [61]. Similarly, CD4+ CD56+ lymphocytes were reduced in post-transplant HCV patients who showed severe post-operative recurrence of infection [62]. Treatment of HCV patients with pegylated IFN-alpha resulted in multiple changes in NK cells, consistent with their activation in vivo, including CD16 expression and cytotoxic function [63]. Several papers suggest that NK cell activity predicts the clinical response. Thus, markers of NK cell immunity, including phosphorylation of STAT1 and TNF-related apoptosis-inducing ligand (TRAIL) expression in NK cells, were predictors of a good response to DAA treatment [64].

Markers of NK cell activation also correlated with strong T cell responses, whether or not the virus was eliminated [65]. The low expression of NK cell inhibitory receptors was a particularly good marker for effective NK immunity and virus clearance [66]. Conversely, a high expression of the NK cell activating receptor NKp46 was associated with the ability to suppress HCV replication in Huh7 cells in vitro [67]. Moreover, in DAA-cured patients, an enhanced IFN signature is observed at baseline in liver and blood [64], and NK cell phenotype and function may be normalized following DAA cure of infection [68]. Thus, at both the level of IFN responses and NK cells, there is intense cross-talk between the innate immune system and HCV infection.

There is an emerging literature looking at immune restoration of T cell functions following DAA-mediated cure. For example, regulatory T cell (Treg) frequencies remain high post-DAA cure, and as such, may hinder immune surveillance of HCC [69]. For mucosal-associated invariant T (MAIT) cells, virus infection, including by HCV, activates these cells [70]. Recent studies have shown that chronic HCV is associated with decreased intrahepatic MAIT cells, while DAA treatment of HCV infection decreases intrahepatic MAIT cell activation and cytotoxicity and increases the MAIT cell frequency [71]. However, not all studies find that HCV-induced MAIT cell dysfunction is corrected after DAA therapy, in both HCV infection [39] and in HCV/HIV infection [72]. Clearly, additional studies are warranted to better understand the T cell phenotype, function, and correlates with liver disease progression in the DAA-cured liver.

## 4. HCV Induces Epigenetic, Gene Expression, and Signal Transduction Abnormalities Associated with Liver Cancer Risk, and These Abnormalities Persist Following DAA Cure of Infection

### 4.1. Epigenetic Changes

Using transcriptomic and epigenomic technologies [73,74,75,76,77,78], it has been shown that HCV infection induces genome-wide epigenetic changes that reprogram host gene expression, and these virus-induced changes persist as an “epigenetic memory” [79] or “epigenetic scarring” [78] following virus eradication by DAA treatment. The results are based on multiple experimental approaches, including human hepatoma cells [77,79,80], viable human liver slice cultures [80,81], human liver chimeric mice engrafted with primary human hepatocytes for infection with HCV and DAA cure [78,82,83], human liver tissues [78], and 3D pre-neoplastic and HCC spheroids from patients with DAA cure [84]. DAA-cured liver tissue from patients with advanced liver disease (i.e., fibrosis scores of F4) and human hepatoma Huh7.5.1 cells cured by DAAs have a different epigenetic profile (i.e., histone H3 protein at a lysine amino acid at position 27 (H3K27ac) that is associated with open and transcriptionally active chromatin [82]) than HCV-infected or non-infected subjects with mild to moderate liver disease or HCV-infected vs. DAA-cured cells. Similar results were obtained using a mouse model [79].

Two independent laboratories have shown that chemical or genetic modulation of histone-modifying enzymes causes reversal of HCV-induced epigenetic changes. For example, treatment of HCV-infected cells with the histone acetyl transferase (HAT) p300/CBP inhibitor C646, which inhibits acetylation of H3K9, or suberoylanilide hydroxamic acid (SAHA), which blocks histone deacetyltransferases (HDAC) of class I and II activity, or knockdown of bromodomain proteins BRD3, BRD4 and histone deacetylase 9 (HDAC9), reverted the HCV-induced changes in the HCV cell culture model [78,84].

### 4.2. Gene Expression and Signaling Changes

The effects of these epigenetic changes on the status of “the prognostic liver signature” (PLS) were then analyzed. The PLS is a well-characterized, 186-gene liver expression signature that has been shown to predict survival and HCC risk in patients with advanced liver disease of all etiologies [83]. The PLS comprises 73 genes that are up-regulated in patients with low survival and HCC (defined as “poor prognosis/high HCC risk genes”) and 113 genes that are up-regulated in patients with high survival and do not have HCC (defined as “good prognosis/low HCC risk genes”). In line with a previous study [83], genes that were epigenetically altered were also transcriptionally altered, predicting high risk of HCC in non-alcoholic steatohepatitis (NASH), CHC, and DAA-cured patients with established HCC as well as in NASH and HCV-infected cell culture models [79].

Bioinformatics analyses reveal that the PI3K pathway is epigenetically perturbed upon chronic HCV infection in patients, and that PI3K pathway activation persists after DAA eradication of the virus. The PI3Ks are associated with transmembrane receptors for cytokines and growth factors including G-protein coupled receptors (GPCRs) and receptor tyrosine kinases (RTKs). PI3Ks are found on the cytoplasmic side of the plasma membrane. Receptor activation by cytokines or growth factors activates PI3Ks to catalyze the phosphorylation of lipid substrate phosphatidylinositols (PIs) into phosphatidylinositol (3,4,5) tri-phosphates (PIP3). PIP3 molecules in turn activate downstream signaling kinases, including PKB and mammalian target of rapamycin (mTOR), which promote cell growth and survival [85]. PI3K activation is often elevated in liver disease, including fibrosis [86,87] and HCC [88]. These data suggest that pathways like PI3K are activated by virus infection and remain “on” after the virus is eliminated by DAAs. Discovery of pathway changes like these may reveal mechanisms that account for the persisting risk of liver disease in patients who are cured of their HCV infection.

**Figure 1:**
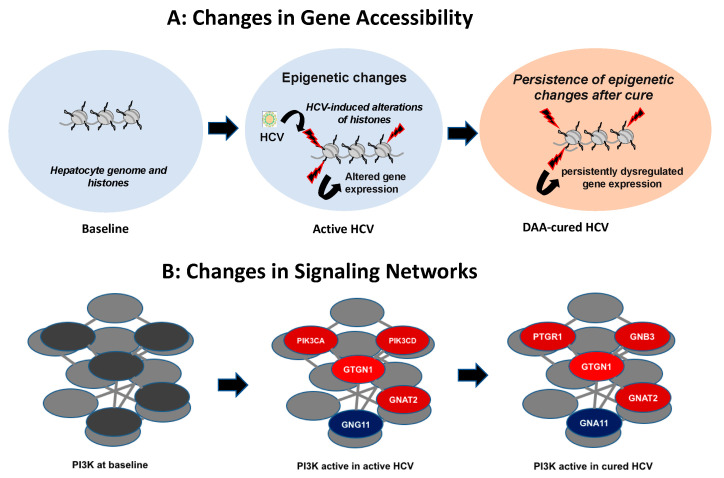
HCV-induced epigenetic and signaling changes persist following DAA-induced cure of infection. (**A**) Schematic of how the baseline epigenetic status is altered by HCV infection, and these alterations persist following cure of chronic infection by DAAs. (**B**) Schematic of a subset of the PI3K signaling network where HCV induces (in red) or suppresses (in blue) components of PI3K signaling, and these changes persist following cure of HCV infection. Gene names are from https://www.genecards.org/.

As a specific example of a gene alteration, the protein sphingosine kinase 1 (SPHK1) level was induced by HCV infection and remained elevated after DAA cure [79]. SPHK1 catalyzes the phosphorylation of sphingosine to form sphingosine-1-phosphate (S1P), which regulates proliferation and survival intracellularly; extracellularly is a ligand for cell surface G protein-coupled receptors. SPHK1 is also a PI3K regulated gene [89]. In a cohort of patients with HCV cirrhosis (n = 216), among which a subset of patients achieved cure (n = 21), high expression of SPHK1 was significantly associated with HCC risk in both cohorts (*p* < 0.034 for HCV cirrhosis and *p* < 0.006 for sustained virologic response (SVR), i.e., cure cohorts) [79]. Thus, SPHK1 may be a potential predictor of HCC risk post-SVR. SPHK1 induction by HCV and persistence following DAA cure of HCV infection have also been observed in Huh7-DAA cell culture cure experiments (unpublished data). Furthermore, we have found similar hepatic alterations that persist in liver slice cultures from DAA-cured liver tissue [81]. Thus, persisting epigenetic changes lead to transcriptional and gene expression changes that are associated with the risk and development of HCC in DAA-cured patients (Figure 1).

## 5. Conclusions/Clinical Implications

Viral cure by DAAs in the majority of infections in most people at all stages of liver disease is a breakthrough in the management of chronic hepatitis C. Nevertheless, the issue of how to manage cured patients with advanced liver disease and how to mitigate the risk of HCC is only beginning to be understood. Recent studies indicate that HCV-induced epigenetic, signaling, and gene expression changes persist after virus eradication and are strongly correlated with a risk of developing HCC. These virus-induced alterations that persist after virus eradication also extend to the level of hepatic innate immunity and hepatic non-parenchymal cells. Continued research is required to better define the molecular mechanisms of action by which HCV induces persistent epigenetic changes and how these changes confer the reprogramming of cellular gene expression and signaling pathways that leads to liver cancer. These questions require research structures that use multiple, complementary experimental systems that range from cells to tissues to in vivo models. The discovery of persisting epigenetic, transcriptomic, and signaling alterations involved in post-HCV immunopathogenesis may represent clinically actionable targets for urgently needed HCC chemoprevention, completing the comprehensive care of people who are cured of their HCV infection. In addition, defining markers for liver disease progression occurring in spite of the cure of HCV could provide valuable diagnostic tools for clinicians.
